# The Medical Annual

**Published:** 1914-06

**Authors:** 


**The Medical Annual.** Bristol: John Wright & Sons Ltd. 1914. 8s. 6d.

The past year having been one of exceptional activity in all departments of medical science, the thirty-second volume contains about one hundred extra pages. Every effort is made to make the articles as concise as possible, and so keep the volume from growing to an inconvenient size. The cosmopolitan character of the work enables the publishers to obtain the assistance of the leaders of medical thought and research in all parts of the world. The pages of the glossary of new terms will be found useful, and the editor's table contains a vast amount of information on the newest products and devices known to the most advanced scientist. Even that interesting combination of microscope and telescope called the "Davon" patent finds a place. The volume continues to be a *vade mecum*

for the consulting-room table, and has become a necessary part of its equipment.

